# Correction: Development and characterization of three-dimensional antibacterial nanocomposite sponges of chitosan, silver nanoparticles and halloysite nanotubes

**DOI:** 10.1039/d4ra90090a

**Published:** 2024-09-02

**Authors:** A. Hernández-Rangel, P. Silva-Bermudez, A. Almaguer-Flores, V. I. García, R. Esparza, Gabriel Luna-Barcenas, C. Velasquillo

**Affiliations:** a Instituto Politécnico Nacional, ESIQIE Av. IPN S/N Zacatenco Mexico City 07738 Mexico adhernandezra@ipn.mx; b The Institute of Advanced Materials for Sustainable Manufacturing, The School of Engineering and Sciences, Tecnologico de Monterrey Campus Queretaro Epigmenio González 500 Querétaro Qro. 76130 Mexico gabriel.luna@tec.mx; c Unidad de Ingeniería de Tejidos, Terapia Celular y Medicina Regenerativa, Instituto Nacional de Rehabilitación Luis Guillermo Ibarra Ibarra 14389 Ciudad de México Mexico mvelasquillo@inr.gob.mx; d División de Estudios de Posgrado e Investigación, Facultad de Odontología, Universidad Nacional Autónoma de México 04510 Ciudad de México Mexico; e Centro de Física Aplicada y Tecnología Avanzada, Universidad Nacional Autónoma de México Boulevard Juriquilla 3001 Santiago de Querétaro 76230 Mexico

## Abstract

Correction for ‘Development and characterization of three-dimensional antibacterial nanocomposite sponges of chitosan, silver nanoparticles and halloysite nanotubes’ by A. Hernández-Rangel *et al.*, *RSC Adv.*, 2024, **14**, 24910–24927, https://doi.org/10.1039/D4RA04274C.

The authors regret that the name of one of the authors (Gabriel Luna-Barcenas) was shown incorrectly in the original article. In addition, Gabriel Luna-Barcenas' affiliation was incorrectly given. The corrected author name and affiliation are as shown above.

The Royal Society of Chemistry apologises for these errors and any consequent inconvenience to authors and readers.

